# Prevalence of enteric bacterial pathogens in diarrheic under-five children and their association with the nutritional status in Bahir Dar Zuria District, Northwest Ethiopia

**DOI:** 10.1186/s40795-023-00678-0

**Published:** 2023-02-24

**Authors:** Mastewal Balew, Mulugeta Kibret

**Affiliations:** College of Science, Department of Biology, Bahir Dar University, Bahir Dar, Ethiopia

**Keywords:** Diarrhea, Under-five children, Nutritional status, Enteric bacterial pathogens

## Abstract

**Background:**

Diarrheal disease is one of the leading causes of child mortality in low and middle-income countries. Low nutritional status and bacterial infections contribute to growth deficiency and death in children. But there is a gap in identifying the bacterial etiology of diarrheal diseases and their association with the nutritional status of under-five children. This study aimed to determine the bacterial etiology of diarrheal diseases and their association with the nutritional status of diarrheic under-five children.

**Methods:**

A cross-sectional study was carried out from February 2021 to March 2022 at seven Health Centers in Bahir Dar Zuria district, Ethiopia. A total of 196 diarrheic under-five children visiting the health centers were included in the study. Stool samples were collected from each child for the isolation of *Salmonella*, *Shigella*, and *E.coli* O157:H7. The demographic characteristics and symptoms of children were obtained from parents/guardians. The weight, height, and age of each child were recorded and anthropometric indices were determined by WHO Anthro version 3.2.2 software. The association between bacterial prevalence and the nutritional status of children was analyzed by SPSS version 26 software using Binary logistic regression. All analyses were conducted at a 95% confidence interval and significant association was determined using a *p*-value < 0.05.

**Results:**

Of the total children included in the study, 13.1% had either *E*.*coli* O157:H7, *Shigella*, or *Salmonella*. Watery diarrhea and fever were the most clinical characteristics observed in children who are positive for enteric bacteria. The prevalence of stunted, underweight, and wasted was 56.6%, 24.4%, and 13.2% respectively. Children with wasting were significantly associated with *Salmonella* detection (OR = 7.2, CI, 1.38–38.1, *P* = 0.02) whereas stunted and underweight were not associated with bacterial prevalence.

**Conclusion:**

Overall, the prevalence of bacterial pathogens in the study area is high. Stunting, wasting, and being underweight are important nutritional deficits of diarrheic under-five children in the study site. Further studies targeting possible sources of bacteria and determinants of malnutrition in children are suggested. Health sectors found in the district should increase their effort to enhance good nutritional practice through health education and treatment of malnourished children by the provision of micronutrients.

## Background

Diarrhea is defined as the passage of loose or liquid stools three or more times per day. It is a symptom of infection in the intestinal tract which is caused by a variety of bacterial, viral, and parasitic organisms [1]. Both diarrhea and pneumonia are responsible for an estimated 40% of all child death around the world each year. Among an estimated 2.5 billion cases of diarrhea that occur among under-five children each year, more than half of these cases are in Africa and South Asia [2].

In Ethiopia, diarrheal diseases are a major health problem in under-five children. The Ethiopian Demographic and Health Survey Report of 2016 shows that 12% of under-five children had diarrhea episodes in the two weeks before the survey. Even though interventions are made to reduce under-five mortality related to diarrhea, the problem remains a major concern [3].

The major causes of diarrheal diseases in under-five children are infectious pathogens including viruses, bacteria, and parasites [4]. In Ethiopia, the prevalence and drug resistance pattern of gram-negative enteric bacterial pathogens from diarrheic patients shows that there is a high burden of infection with gram-negative bacterial pathogens. The Amhara National Regional State is the second with a high prevalence of gram-negative enteric bacterial pathogens like *Escherichia coli*, *Campylobacter* spp., *Shigella* spp., and *Salmonella enterica* [5].

Malnutrition is an important factor that determines the recurrence of diarrheal diseases and child mortality [6]. Globally, 150.8 million and 50.5 million of under-five children are stunted and wasted respectively. Even though stunting among children declined from 198.4 million to 150.8 million globally, in Africa the actual number has risen due to population growth [7].

Chronic malnutrition or stunting is more common in Ethiopia than in surrounding African countries. Recently there is an increase in chronic malnutrition and the prevalence of under nutrition remains very high [8]. Ethiopian demographic and health survey report of 2011 shows, 44% of under-five children are stunted, and 21% of children, are severely stunted. One in ten under-five children is wasted. Almost 30% of children under the age of five are underweight. In resource-limited areas of Ethiopia, stunting and being underweight have a significant relation with the overall delay of childhood development [9].

Nationally, the prevalence of stunting, wasting, and underweight shows decrement from 2005 to 2011. But in Amhara National Regional State, stunting increased from 52% in 2005 to 56% in 2011. Wasting increased from 9.9% in 2005 to 14.2% in 2011 and underweight increased from 33.4% in 2005 to 48.9% in 2011 [10]. Frequent research is required to identify the risk factors of undernutrition among children in Ethiopia [8]. There is a need for district-specific policies to reduce the high risk of stunting [11].

Enteric infections canlead to chronic gut inflammation and reduced absorptive capacity for nutrients. Beyond the poor nutrition characteristics of diets, the role of recurrent or persistent enteric infections on the nutritional status of children is poorly known [12]. There are research reports on the high prevalence and burdens of diarrheal diseases in the study area. In reducing the problem, there is a gap in determining the role of recurrent enteric infection for diarrheal diseases and the nutritional status of children. So far no studies have been conducted on the prevalence of enteric infections and their impact on the nutritional status of children in the study area. So in the necessity of conducting this research is to fill the gap in the literature by determining the prevalence of enteric bacterial pathogens and their role in the nutritional status of diarrheic under-five children in Bahir Dar Zuria district, Ethiopia.

## Methods

### Description of the study area

**Fig. 1:**
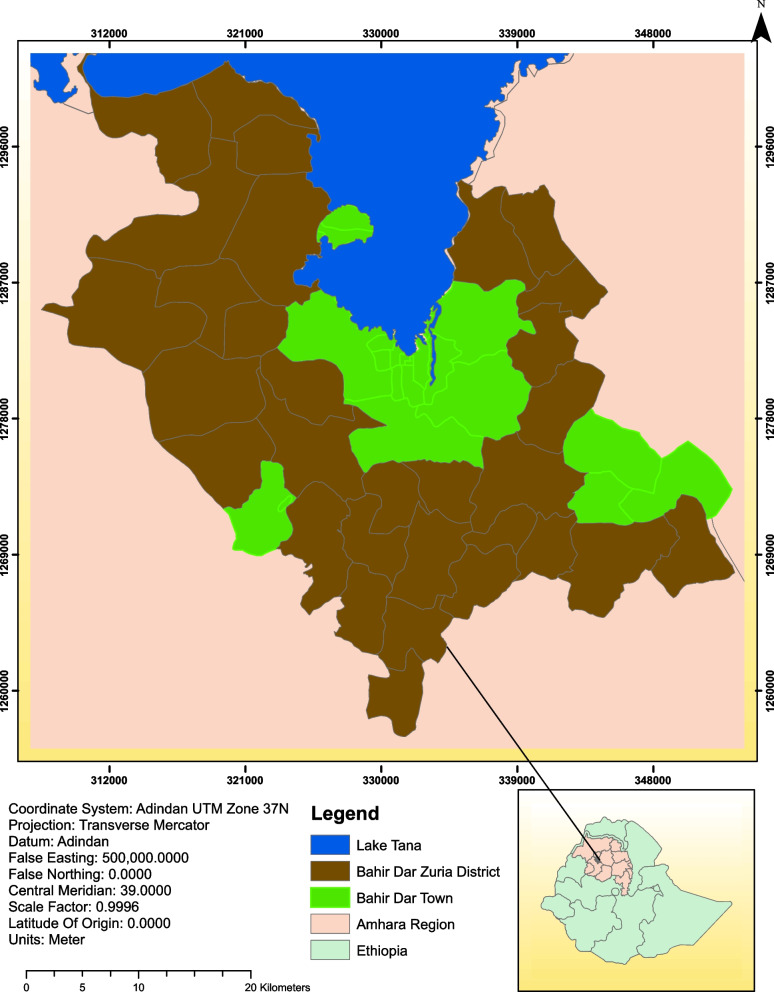
Location of the study area

The study was conducted in Bahir Dar Zuria district, Amhara National Regional State, Ethiopia to determine the prevalence of enteric bacterial pathogens and their association with the nutritional status of diarrheic under-five children. Bahir Dar Zuria district is one among 15 districts found in West Gojjam Zone, Northwest Ethiopia. It locates in the range from 11^0^17′34" to 11^0^50′05" latitude and 37^0^05′21" to 37^0^39′14" longitude with an area of 1,443.37 square kilometers (Fig. 1). Bahir Dar Zuria district has a total population of 283,514 [13].

### Study design and period

A cross-sectional study was conducted to determine the prevalence of enteric bacterial pathogens and their association with the nutritional status in 196 diarrheic under-five children. The study was conducted in Bahir Dar Zuria district from February 2021 to March 2022 in seven health centers found in Bahir Dar Zuria district namely Kinbaba, Yinesa, Robit, Addis Alem, Wonjata, Han, and Shinbet Health Centers.

### Sample size determination

The number of diarrheic under-five children included in the study was calculated by a single population proportion formula with a 95% confidence interval and 5% of marginal error. The formula used for sample size calculation was N = 1.96^2^ P (1-P)/d^2^: Where N is the sample size, P is the expected prevalence or proportion, and d is precision or margin of error [14]. By using the 13.1% average prevalence of bacterial pathogens (*Salmonella*, *Shigella*, and *E*.*coli* O157:H7) among under-five children with acute diarrhea in Bahir Dar city reported previously [15], with the assumption of a 5% expected margin of error and considering a 95% confidence interval the calculated number of under-five children with diarrhea included in the study was 196.

### Sampling techniques and stool sample collection

Seven health centers found in the Bahir Dar Zuria district were sites for sample and data collection. Under-five children who had diarrhea and got care at those seven selected health centers were included in the study. Stool samples were collected by the researcher from each under-five child with diarrhea coming to selected health centers for treatment during the study period. Two to five grams of freshly passed stool samples were collected aseptically by using a sterile stool cup from each diarrheic under-five child. The stool samples were transported to Bahir Dar University Microbiology Laboratory by using an icebox and processed within six hours of collection.

### Anthropometric and socio-demographic data collection

Socio-demographic and clinical data of the study participants were collected through interviews with parents/guardians of children. The height and weight of each diarrheic under-five child who comes to selected health centers for treatment were recorded by skilled health professionals. WHO Anthro software was used for determining the anthropometric indices. Anthropometric indices (Height for Age, Weight for Height, and Weight for Age) were calculated by using WHO Anthro version 3.2.2 software. The Anthropometric indices were used to determine the nutritional status of each child and categorize them into stunted, underweight, and wasted based on WHO child growth standards [16]. For anthropometric data analysis, children whose height for age Z score is below minus two standard deviations (-2 SD) from the median of the reference population are considered as stunted. If the weight for height Z score is below minus two standard deviations (-2SD) from the median of the reference population, the child is wasted. Children whose weight for age Z-score is below minus two standard deviations (-2SD) from the median of the reference population are considered as underweight [17].

### Inclusion and exclusion criteria

Under-five children with diarrhea who comes to selected Health Centers for treatments during the study period were included in the study. Children who had treatment with any antibiotics within two weeks before the time of data collection were excluded from the study.

### Isolation of Salmonella, Shigella and E.coli O157:H7

To isolate *Salmonella*, *Shigella*, and *E.coli* O157:H7 from under-five children, enrichment and selective media for each pathogen were used and standard laboratory protocols were followed. Based on the morphology and color of colonies, suspected *Salmonella*, *Shigella,* and *E.coli* O157:H7 isolates were further identified by using a series of biochemical tests [18]. To isolate *Salmonella* and *Shigella* from under-five children, each stool sample was first inoculated into selenite F enrichment broth (HiMedia, India) and incubated at 37 °C for 18- 24 h. Then from the selenite F enrichment broth, a loopful of culture was sub-cultured onto xylose lysine deoxycholate agar (HiMedia, India) and Salmonella-Shigella agar (HiMedia, India) and then incubated at 37 °C for 24 h. Suspected *Salmonella* and *Shigella* colonies were detected by their color and morphology on xylose lysine deoxycholate agar and Salmonella-Shigella agar. Red colonies and red colonies with black centers on XLD agar were suspected colonies of *Shigella* spp. and *Salmonella* spp. respectively*.* Colorless colonies and colorless colonies with black centers on *Salmonella Shigella* agar were isolated as *Shigella* spp. and *Salmonella* spp. Respectively [19]. The *Salmonella* and *Shigella* isolates were further identified by a series of biochemical tests.

To isolate *E.coli* O157:H7 from under-five children**,** the stool sample was first diluted to sterile saline solution and inoculated onto Sorbitol MacConkey (SMAC) (HiMedia, India), and incubate at 37^0^C for 18–24 h. Sorbitol-negative colonies appear colorless on Sorbitol-MacConkey Agar. Since *E.coli* O157:H7 is a sorbitol non-fermenter (Sorbitol negative), they appear colorless on Sorbitol-MacConkey Agar. Colorless colonies on Sorbitol MacConkey Agar were suspected colonies of *E.coli* O157:H7 and were confirmed by further biochemical tests [20].

### Biochemical identification of Salmonella, Shigella, and E.coli O157:H7 isolates

The suspected colonies of *Salmonella* and *Shigella* isolates were further identified biochemically using Gram staining, Triple Sugar Iron Agar test, Urease test, Citrate test, Lysine decarboxylase test, Motility, and Indol tests [19]. *E*.*coli* O157:H7 were confirmed by IMViC test (Indol, Methyl red, Voges Proskauer, and Citrate utilization) tests [20].

### Data analysis

Data were analyzed by using Statistical Package for Social Sciences (SPSS) version 26 software. In data analysis, first descriptive statistics were used to describe the Socio-demographic characteristics of respondents. Binary logistic regression was used to determine the association between the nutritional status of children and the prevalence of *Salmonella*, *Shigella*, and *E.coli* O157:H7. *P* < 0.05 were considered a significant association.

## Results

### Socio-demographic characteristics of the study participants

Among a total of 196 diarrheic under-five children included in the study, more than half of the participants were from Kinbaba health center. Of the total children included in the study, 100(51%) were males and 96(49%) were females. Most of the study participants 191(97.4%) were rural residents (Table 1).

**Table 1:** Socio-demographic characteristics of the study participants

Variables	Categories	Frequency	Percentage
Health centers	Kinbaba	105	53.5
	Yinesa	52	26.5
	Han	25	12.7
	Addis Alem	3	1.5
	Shinbet	5	2.5
	Robit	1	0.5
	Wonjata	5	2.5
Gender	Male	100	51
	Female	96	48.9
Residence	Urban	5	2.6
	Rural	191	97.4
Domestic animals in the household	Yes	196	100
	No	0	0

### Prevalence of Salmonella, Shigella, and E.coli O157:H7 in diarrheic under-five children

**Fig. 2:**
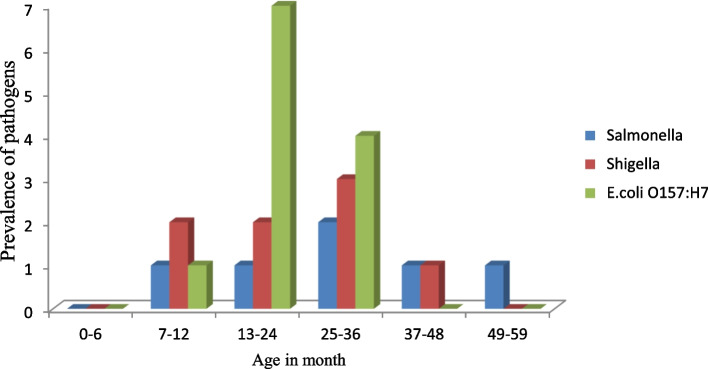
Prevalence of bacterial isolates in diffrent age groups

*E*.*coli* O157:H7, *Shigella*, and *Salmonella* were detected from 12(6.1%), 8(4%), and 6(3.1%) samples respectively. From a total of 26 bacterial isolates, 11(42.3%) were detected from males, and 15(57.6%) were detected from females. No significant difference was observed in the prevalence of *E*.*coli* O157:H7, *Shigella*, and *Salmonella* between males and females. There was a variation in the prevalence of *Salmonella*, *Shigella*, and *E.coli* O157:H7 among different age groups. *Shigella* and *E*.*coli* O157:H7 were not detected in children below six months and between 48 and 59 months of age groups but a relatively large number of *Shigella* and *E*.*coli* O157:H7 were detected between age groups 13 months and 36 months (Fig. 2).

#### Nutritional status of children

**Fig. 3:**
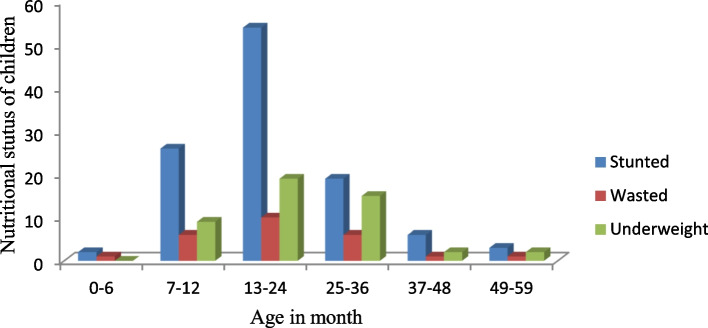
The nutritional status of children in diffrent age categories

Based on the anthropometric indices obtained from Anthro software, the prevalence of stunted, wasted, and under-weight children were determined. The prevalence of stunted, underweight, and wasted in diarrheic children was 111(56.6%), 48(24.4%), and 26(13.2%) respectively. There was no significant variation in the nutritional status between males and females. The nutritional status of children varies in different age ranges. Among the three nutritional groups, there was a significant difference in stunting among different age groups (chi-square, X^2^ = 0.004). A significantly higher number of stunted children were observed at the age range between 13 and 36 months than others (Fig. 3).

#### Clinical characteristics of diarrheic children

The most common clinical complaints associated with diarrheal diseases in under-five children were vomiting 129(65.8%) and watery diarrhea 128(65.3%). In contrast, bloody diarrhea was observed in only three diarrheic children. Among the clinical characteristics recorded from diarrheic children who are positive for enteric bacterial pathogens, watery diarrhea 15(57.6%) and fever 9(34.6%) were mostly observed in children positive for *Salmonella*, *Shigella*, and *E.coli* O157:H7. Bloody and loose diarrhea was observed in very few samples (Table 2).

**Table 2:** Clinical characteristics of children who are positive for *Salmonella*, *Shigella*, and *E*.*coli* O157:H7

Characteristics	Type of bacterial isolate			
	*Salmonella* Spp	*Shigella* Spp	*E*.*coli* O157*:*H7	Total (%)
Watery diarrhea	4	4	7	15(57.6)
Bloody diarrhea	0	0	2	2(7.6)
Mucoid diarrhea	2	3	2	7(26.9)
Loose diarrhea	0	1	1	2(7.6)
Fever	4	3	2	9(34.6)
Vomiting	3	1	2	6(23)

### Association between the prevalence of bacteria and nutritional status of children

The association between pathogen prevalence and nutritional status was analyzed by using binary logistic regression. More *Salmonella* and *E.coli* O157:H7 was detected in stunted children than in non-stunted ones. But there was no significant difference between the prevalence of *Salmonella* and *E*.*coli* O157:H7 between stunted and non-stunted children. On the other hand, more *Shigella* and *E.coli* O157:H7 have been detected in non-wasted and non-underweighted children. Among the three nutritional categories, only being wasted was significantly associated with *Salmonella* prevalence (*P *= 0.02). The remaining two (stunted and underweighted) are not associated with the prevalence of either of each pathogen (Table 3).

**Table 3:** Association between the nutritional status of children and the prevalence of bacteria in under-five children

Detected bacterial pathogen	Stunted		OR(95%CI)	*P* value	Wasted		OR(95%CI)	*P* value	Under weight		OR(95%CI)	*P* value
	Yes	No			Yes	No			Yes	No		
*Salmonella*spp.	4	2	1.5(0.27–8.6)	0.61	3	3	7.26(1.38–38.1)	0.02*	3	3	3.22(0.62–16.5)	0.16
*Shigella* spp.	4	4	0.75(0.18–3.11)	0.7	0	8	0(0)	0.99	1	7	0.42(0.05–3.57)	0.43
*E*.*coli* O157:H7	7	5	1.07(0.33–3.51)	0.9	1	11	0.57(0.07–4.67)	0.6	4	8	1.59(0.45–5.53)	0.46

^*^significant association

## Discussion

The overall prevalence of *Salmonella*, *Shigella*, and *E*.*coli* O157:H7 obtained in the current study was 13.1%. This result is comparable with the previous study conducted in 2017 in Bahir Dar city, Ethiopia which shows the prevalence of *Salmonella*, *Shigella*, and *E*.*coli* O157:H7 was 13.1% [15]. The prevalence rate of *Salmonella* and *Shigella* in the current study is greater than previously reported in 2014 in Ambo town, Ethiopia which was 3.8% [21]. *Shigella* prevalence obtained in this study is also higher than reported in Nekemtie, Oromia region, Ethiopia between October 2015 to February 2016 [22], and in Jimma, Southwest Ethiopia which was obtained in 2012 [23], the prevalence was 2.2% and 2.3% respectively. Unlike the participants involved in studies conducted in Nekemtie and Jimma, almost all participants (97.4%) involved in the current study were rural residents. Being a rural resident contributes to diarrheal diseases and infection with causative agents due to lower access to pure drinking water, toilet availability, and low personal and environmental hygiene in rural areas than urban areas. This could contribute to the higher prevalence observed in the current study than in the studies conducted in Ambo town, Nekemtie, and Jimma [21–23].

Contrarily, the prevalence of *Salmonella*, *Shigella*, and *E.coli* O157:H7 in the current study is lower compared to the pooled prevalence of *Salmonella*, *Shigella*, and *E.coli* O157:H7 observed in eight regions of Ethiopia reported in 2021 which was 6.24%, 5.06%, and 19.7% for *Shigella*, *Salmonella,* and *E.coli* O157:H7 respectively [5]. In addition, the prevalence of *Salmonella* observed in the current study is lower compared to reported in Robe, South East Ethiopia in 2016 [24], between October 2015 to February 2016 in Nekemtie [22], in 2012 in Addis Ababa [25], and in 2017 in Arba Minch, south Ethiopia [26] in which the prevalence was 6.9%, 7%, 10%, and 17.4% respectively. The prevalence of *Shigella* in the current study was also lower than reported in 2011 in Hawassa, South Ethiopia (7%) [27] and Gondar, Northwest Ethiopia in 2018 (10.7%) [28]. The lower prevalence of *Salmonella*, *Shigella*, and *E*.*coli* O157:H7 could be due to different factors including, variation of socio-demographic and environmental characteristics, methods used in isolation of pathogens, and the presence of other potential enteric bacterial, viral, and parasites causing diarrhea.

In this study, the prevalence of *Salmonella*, *Shigella* and *E*.*coli* O157:H7 varies in different age groups. A higher prevalence of *Salmonella*, *Shigella*, and *E*.*coli* O157:H7 was observed between age groups 13 months and 36 months and a lower number was observed in children aged less than six months and between 49 to 59 months. This result was supported by a study conducted in 2014 in Ambo town, Ethiopia [21] and in Robe, South East Ethiopia conducted in 2016 [24] which reported that children between the age groups 13 months and 36 months are more exposed to *Salmonella* and *Shigella* infections. Most children below six months of age didn’t begin compulsory food and are less exposed to infection than elders. After six months of age when they began compulsory food, they may infect with *Salmonella*, *Shigella*, and *E.coli* O157:H7 through food or water.

In addition to determining the prevalence of *Salmonella*, *Shigella*, and *E*.*coli* O157:H7, the nutritional status of diarrheic under-five children was also determined. A high prevalence of stunting, being underweight, and wasting was observed among children. Of the total children enrolled in the study, 56.6%, 24.4%, and 13.2% were stunted, under-weight, and wasted respectively. The nutritional status of under-five children included in this study shows that more than half of the children are stunted which means they have a lower height for their age than the growth standard set by World Health Organization [17]. It is higher than a study conducted in Ethiopia by using 2016 Ethiopian demographic and survey data to identify the risk factors associated with childhood undernutrition in Ethiopia [29] which showed that 49% of children were undernourished. The prevalence of stunting obtained in this study was higher than the national and regional reports which showed 39% and 46.3% [30] respectively. Similarly, the prevalence of stunted children obtained in this study was higher than reports of different regions of Ethiopia. Studies conducted in 2011 in Hawassa Zuria district, Southern Ethiopia, and the study conducted based on the Ethiopian Demographic and Health Survey report of 2016 in Somali region, Ethiopia shows the prevalence of stunting was 45.8% and 27.4% respectively. The reason for the high prevalence of stunting observed in this study could be, the children included in the current study were only diarrheic, which highly contributes to the occurrences of stunting in children [31]. In addition, since most study participants involved in the current study were rural residents this could contribute to the high prevalence of stunted children. It is supported by a study conducted in Kenya (1998–2009) and Zambia(1996–2014) which shows that there is a high risk of stunting among children living in rural areas and with an overall lower wealth index [32].

A significantly higher number of stunted children were observed between ages 13 and 24 months when children start compulsory food. According to the Ethiopian demographic and health survey report of 2016, only 7% of children aged 6–23 months meet the minimum acceptable dietary standards. Only 14% of children had an adequately diverse diet. This might result in a higher number of stunted children in this age range. It is in line with the Ethiopian demographic and health survey report of 2011 [10] and 2016 [3]. Of the total number of under-five children included in the study, 24.4% are underweight. The prevalence of underweight children obtained in this study was less than the regional reports of 2016 which was 28.4% [33]. Similarly, the prevalence of underweight children in this study was less compared to studies conducted in Hawassa town in 2011 and based on Ethiopian demographic and health survey reports of 2016 in Somali regions were 31.9% [34]and 28.7% [35] respectively, Among the three nutritional categories, the least nutritional status found in the study site was wasting which accounted for 13.2%. Even if wasting is the least malnutrition status among the three nutritional groups in the current study, it is higher than the national and regional report which shows 10% and 9.8% respectively [33]. Overall, the nutritional status of under-five children included in this study shows there is a high prevalence of chronic malnutrition in the study area which needs serious attention to improve the nutritional value of child food.

The association between the prevalence of *Salmonella*, *Shigella,* and *E*.*coli* O157:H7 and the nutritional status of children were also assessed. Stunting and being under-weight were not associated with the prevalence of *Salmonella*, *Shigella*, and *E*.*coli* O157:H7. This study is in line with the study conducted in Kenya between 2011 and 2014 which shows that no significant association was observed between stunting and pathogen detection in diarrheic children [36]. Children with *Salmonella* detection had lower weight for height z scores (WHZ) means wasted. A significant association was observed between *Salmonella* detection and Wasting (*p* = 0.02). A study conducted in Brazil In 2017 shows that internal parasitic infections negatively influence the physical development of children [37]. But wasting was not significantly associated with *Shigella* and *E*.*coli* O157:H7 detection. Contrary to the current study, a post-hoc analysis conducted by Global Enteric Multicenter Study (GEMS) shows that Enteropathogenic *E*.*coli* (EPEC*)* and heat-stable enterotoxin-producing *E*.*coli* (ST-ETEC) have a stronger association with moderate-to-severe diarrhea among children with acute malnutrition (wasted) than among children with better nutritional status [38].

Overall the current study has its limitations. The study was limited to the isolation of only three diarrhea-causing bacterial pathogens. It could be good if other pathogens causing diarrheal diseases including intestinal parasites and viruses were isolated from children. In addition unable to identify the determinant factors of the low nutritional status of children in the study site was another limitation of the study. Having such limitations, the current study did a great job of determining the prevalence of enteric bacterial pathogens and the nutritional status of diarrheic under-five children in Bahir Dar zuria district. The study adds value to the existing literature by showing the association between pathogen prevalence and nutritional status in diarrheic under-five children.

## Conclusion

The prevalence of enteric bacterial pathogens *e* in diarrheic children in the study area was high. Since they are the potential causes of diarrhea, avoiding the possible sources is essential for better child health. The nutritional status of diarrheic under-five children in the study area is low. The high prevalence of chronic malnutrition in children needs attention to improve the nutritional value of child food. Even though stunted and wasted children were more affected by *Salmonella* infection, only wasted was significantly associated with *Salmonella* detection. Further studies on the possible sources and factors that contributes to low nutritional status in children are recommended. The finding of this study emphasizes the need for special consideration for the most vulnerable children through the supplement of minerals and vitamins, and awareness creation for the parents on child feeding to improve their nutritional status. Designing and implementing community-based nutrition interventions should be given attention to reduce under nutrition in the district.

## Data Availability

The data sets used and analyzed in the study are available from the corresponding author and provided for a reasonable request.
